# Rosario: An algorithm to analyse cyclical data in Ecology

**DOI:** 10.3897/BDJ.14.e176358

**Published:** 2026-06-12

**Authors:** Tatiana Velásquez-Roa, Maria A. Hurtado-Materon, Angel L. Robles-Fernández, Iván Castro-Arellano

**Affiliations:** 1 Integrative Ecology Lab, Biology Department, Texas State University. 601 University Dr., Texas, 78666., San Marcos, United States of America Integrative Ecology Lab, Biology Department, Texas State University. 601 University Dr., Texas, 78666. San Marcos United States of America; 2 Ecology and Evolutionary Biology Program, Department of Ecology and Conservation Biology, Texas A&M University, 534 John Kimbrough Blvd, Texas, 77840., College Station, United States of America Ecology and Evolutionary Biology Program, Department of Ecology and Conservation Biology, Texas A&M University, 534 John Kimbrough Blvd, Texas, 77840. College Station United States of America; 3 The University of Kansas, Takeru Higuchi Hall 2101 Constant Ave., Kansas, 6604., Lawrence, United States of America The University of Kansas, Takeru Higuchi Hall 2101 Constant Ave., Kansas, 6604. Lawrence United States of America

**Keywords:** diel activity patterns, null model, niche overlap, niche partitioning, phenology, seasonality.

## Abstract

Temporal niche overlap can be effectively studied using null models, which are statistical tools that randomise ecological data to reveal underlying patterns. The Rosario R package enables robust analysis of cyclical phenomena in ecology through the implementation of the Rosario algorithm. The Rosario package preserves temporal autocorrelation (the tendency for events close in time to be similar) and generates biologically realistic time-use scenarios. To ensure continued usability and standardisation, we are transitioning the Rosario algorithm from Windows-based software to the R programming environment. This transition enhances accessibility and compatibility with R packages, such as activity and overlap. Unlike these packages, rosario supports concurrent overlap analysis amongst multiple biological identities (e.g. individuals, species, populations, communities) at the same time, offering a more comprehensive approach to studying cyclical data. Now, users can access rosario in R and work with a full suite of packages in one language, facilitating complementary insights into natural temporal patterns.

## Introduction

Understanding the co-existence of species at local scales and patterns of resource partitioning is central in ecology. Niche dimensions critically influence the co-existence of individuals and populations within communities (Chase and Leibold 2003), with the most significant dimensions being habitat, food resources and time (Pianka 1969). Time is the resource that has received least attention (Gotelli and Graves 1996), despite the fact that time partitioning enables each species to gain exclusive access to resources (Kronfeld-Schor and Dayan 2003). How species use available resources determines the type of partitioning observed. Species may exhibit several forms of niche differentiation, including temporal partitioning (e.g. Guerra et al. (2020), Blašković et al. (2022), Cordero et al. (2024)), trophic partitioning (e.g. Carvalho et al. (2014), Vilela et al. (2017), Nieto et al. (2023)) and spatial partitioning (e.g. Ramesh et al. (2012), Rocha et al. (2019), Bashir et al. (2023)). Access to resources during specific parts of the diel cycle (24-hour day-night period) can be a determining factor for survival (Kronfeld-Schor and Dayan 2003). Together, these ecological mechanisms could facilitate species co-existence and promote local biodiversity.

The use of null models provides an objective framework for detecting non-random patterns in species interactions and resource use (Ulrich and Gotelli 2010). Thus, an effective method to investigate temporal partitioning is the incorporation of null models. A null model is defined as a framework that generates patterns through the randomisation of ecological data from a known or theoretical distribution (Gotelli and Graves 1996). For non-sequential resources, such as food or microhabitat (not ordered in time), simple randomisation models are appropriate because the data exhibit no temporal autocorrelation (i.e. dependence on nearby events in time) (Sale 1974, Lawlor 1980, Winemiller and Pianka 1990). Sequential and cyclical data, such as activity patterns, require specialised randomisation models. Cyclical data refer to observations that repeat over a closed and continuous cycle, where the end point connects back to the beginning (e.g. 24-hour diel cycles, tidal cycles or annual phenological cycles). In such datasets, the temporal variable forms a circular structure with strong autocorrelation between adjacent time intervals. In contrast, sequential data follow a linear progression with a true beginning and end. The Rosario randomisation algorithm by Castro-Arellano (Castro‐Arellano et al. 2010) was specifically designed for cyclical data and is not intended for sequential datasets. However, long-term camera-trap datasets can be analysed with Rosario when time-stamps are mapped on to any cyclical temporal domain (e.g. time of day, season or year), thereby producing a circular activity pattern suitable for the algorithm.

Assessing temporal niche overlap is essential to understanding species ecology and their interactions. Rosario, a randomisation algorithm (Castro‐Arellano et al. 2010), was designed to implement a null model approach to assess temporal niche overlap. Rosario builds on earlier studies (Fleming and Partridge 1984, Tokeshi 1986, Loreau 1989) and uses the Pianka and Czekanowski indices as quantitative measures of similarity in resource use (i.e. time use) (Pianka 1973, Feinsinger et al. 1981) to measure overlap. Rosario preserves the shapes of empirical data by shifting entire patterns across time intervals. Using Monte Carlo simulations, Rosario generates null frequency distributions of cyclical ecological data and can execute thousands of simulations, while maintaining temporal autocorrelation and producing biologically realistic time-use scenarios (Castro‐Arellano et al. 2010). This process differs from other randomisation models, such as scrambled-zeros (RA3) and conserved-zeros (RA4), which handle periods of inactivity (zeros) differently (Sale 1974, Lawlor 1980, Winemiller and Pianka 1990). RA3 and RA4 were developed for non-sequential resource matrices, where the order of resource categories is irrelevant. RA3 randomised resource use within rows while ignoring the structure of zeros, whereas RA4 preserves the position of zeros exactly. As both models treat resource categories as unordered, they disrupt the temporal autocorrelation and circular structure inherent in cyclical data, such as diel or seasonal activity patterns. In contrast, Rosario maintains the empirical shape and autocorrelation of the observed temporal patterns by shifting the entire distribution curve across the cycle, producing biologically realistic null expectations for time-ordered, cyclical datasets. Thus, RA3 and RA4 remain appropriate for non-temporal resource matrices, while Rosario is specifically better suited for applications involving cyclical data (Sale 1974, Lawlor 1980, Winemiller and Pianka 1990).

Rosario was designed within a restricted application framework for analysis of diel temporal overlap within a local ecological community (Castro‐Arellano et al. 2010). However, its potential for a much wider application at larger temporal and spatial scales has been recognised. The input matrix for analysis is not restricted to species by diel time intervals; instead, it can represent individuals, species or populations, tracked over hours, seasons or years. Previously, limited data availability restricted the exploration of these broader applications. However, the recent proliferation of open-access databases like wildlifeinsights.org and movebank.org enables a diverse implementation of this algorithm.

Rosario and similar tools are invaluable in advancing ecological research by providing robust methods for analysing temporal overlap. Since 2010, the Rosario algorithm has been used and is recognised in the academic and research communities. Researchers have used it to analyse diverse taxa, including ants, bats and carnivores (Arriaga-Flores et al. 2012, Brito et al. 2012, Mancina et al. 2012, Ramesh et al. 2012). Insects and mammals are the focus of 25 and 21 studies, respectively, though it has also been applied to birds, anurans and plants (i.e. phenological phenomena). Recent research, such as Silva et al. (2024) on bee resource use and Cordero et al. (2024) on rodent species’ foraging behaviour, highlights Rosario’s versatility and its potential for understanding timing in ecological cycles. The combination of the open source databases and the availability of the algorithm in R likely will trigger a much larger number of contributions to augment our knowledge in this area.

Standardised methodologies in ecological research ensure consistency and comparability across various studies. The Rosario algorithm was initially developed for Windows via the TimeOverlap programme, but Windows updates could eventually make this shareware obsolete. Moving Rosario’s algorithm functionality to the widely used R programming language (R Core Team 2025) will allow its continued accessibility. Currently, other packages, like activity and overlap, measure temporal overlap (Ridout and Linkie 2009, Meredith et al. 2014). However, their analysis is restricted to pairwise comparisons, while Rosario implements a null model approach to evaluate a concurrent overlap amongst multiple biological identities at once, offering a novel analytic perspective in R. These approaches provide a complementary analysis of temporal overlap in ecological settings.

The R environment enables researchers to address scientific questions and integrate packages to improve rigour (R Core Team 2025). By adapting Rosario to R, our aim is to enhance the accessibility and comprehensibility of scientific work. This transition will allow for meaningful comparisons with other R packages (overlap and activity) to analyse activity patterns or more broadly cyclical data. This shift supports the establishment of standardised methodologies for studying temporal niche overlap and, more importantly, enables the inclusion of a null model approach to analyse cyclical data, such as time-stamp images from camera traps.

## Installation

The Rosario package is available on CRAN and can be installed using the standard install.packages() function. The link to the source code repository of the development version is available on GitHub (https://github.com/alrobles/rosario).

# Install the stable version from CRAN

install.packages("rosario")

# Or install the development version from GitHub

# First install devtools if not already installed

install.packages("devtools")

devtools::install_github("alrobles/rosario")

## Usage

Following the original description of the randomisation algorithm (Castro‐Arellano et al. 2010), the package was named Rosario after the tradition rosary praying, in which beads are advanced in a circular fashion analogous of the progression of ecological cycles.

**Figure 1.**
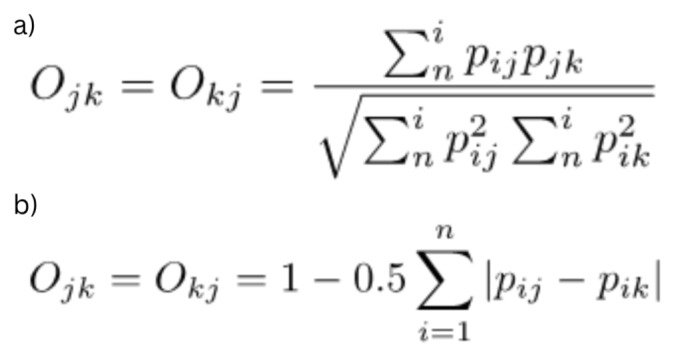
Equations to calculate a) Pianka and b) Czekanowski indices.

The *rosario()* function generates a series of alternative time-use scenarios for each biological identity by sequentially advancing the empirical pattern until the modal value has occupied each of *n* time intervals within the full-time extent. Each alternative time-use scenario represents the same frequency distribution, but with a different location within the time extent. Additionally, *rosario()* includes *m* mirror-images of each one of the previously created time-use scenarios. In each algorithm iteration, a matrix of pseudo time-use scenarios is generated by randomly selecting one of these possibilities from each of the *2n* possibilities of each of *S* biological identities. After creating the matrix of pseudo time-use scenarios, the Rosario package calculates a mean of all pairwise overlaps amongst all biological identities and repeats the process as many times as needed to generate a frequency distribution (e.g. usually 1000 simulations) that is compared against the observed overlap value in the empirical matrix. Rosario works with two indices, Pianka and Czekanowski (Fig. 1) (Pianka 1973, Feinsinger et al. 1981) to analyse temporal overlap.

**Figure 2.**
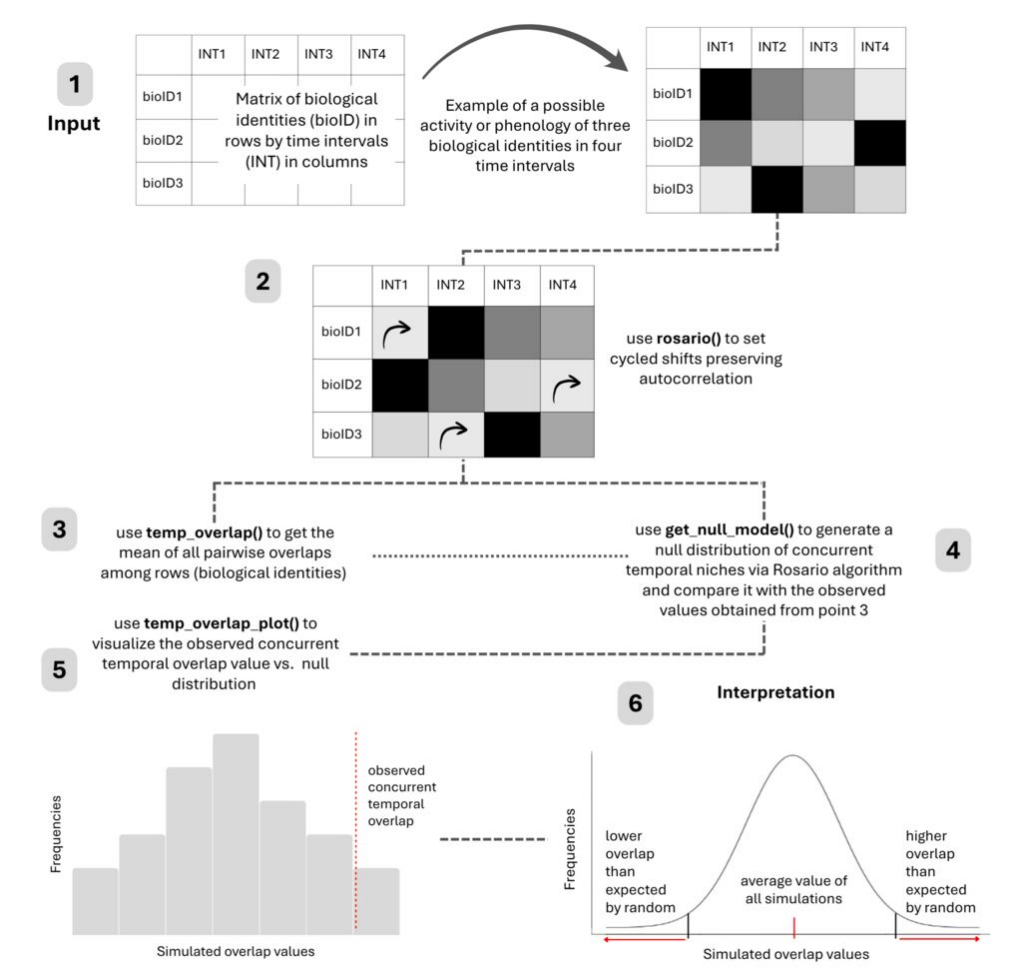
Workflow of the Rosario R package, which implements a null model analysis to quantify concurrent temporal niche overlap (i.e. activity or phenology) amongst biological identities (e.g. individuals, populations, species) using the Rosario randomisation algorithm (Castro-Arellano et al. 2010). In steps 1 and 2, colours represent the intensity of activity, black representing high numbers that show higher activity and white low numbers that show lower activity. In step 6, black line represents the significance threshold of 0.95.

The Rosario package implements a null model analysis to quantify concurrent temporal niche overlap, including activity or phenology, amongst biological identities (e.g. individuals, populations, species) using the Rosario randomisation algorithm (Castro-Arellano et al. 2010). Fig. 2 illustrates the sequential steps of the rosario workflow. Table 1 explains each package function, its main purpose, required inputs and outputs and internal dependencies.

The process begins with a matrix input in which rows represent biological identities and columns represent time intervals. The function *rosario()* generates a set of cyclic shifts and their mirror images (reverse order), maintaining the autocorrelation structure of the empirical data, while changing their temporal location. After generating the complete suite of vectors and mirrors, users can: 1) calculate the mean of all pairwise overlaps amongst rows (biological identities) using *temp_overlap()* function with the chosen index Pianka or Czekanowski; 2) perform a null-model test of concurrent overlap via Rosario algorithm randomisation to obtain the observed value for temporal niche overlap, using *get_null_model()* function; 3) visualize the first 10 hypothetical time use distributions of a single biological identity produced by *plot_rosario()* function. Each panel displays one hypothetical time use distribution with its cyclic shift shown in dark grey and its mirror image shown in dark red; 4) plot the histogram that shows the frequency distributions of the concurrent temporal overlap and the observed value using *tem_overlap_plot()* function.

## Example

To demonstrate the functionality of Rosario, we applied the package to a dataset of cervid activity patterns from the SIM Deer project in British Columbia, Canada, obtained from Wildlife Insights (https://app.wildlifeinsights.org), a publicly accessible dataset (Foster et al. 2020). From the over two million capture events in the SIM Deer project dataset, we focused on data collected during the summer months of June, July and August for the years 2020, 2021 and 2022. The analysis includes three cervid species: Mule deer (*Odocoileus hemionus*), Elk (*Cervus canadensis*) and White-tailed deer (*Odocoileus virginianus*). The dataset comprises a total of 85,929 capture events, with 51,691 events for Mule deer, 19,395 for Elk and 14,843 for White-tailed deer.

For comparative purposes, we used the activity and overlap packages to contrast against the results obtained using the Rosario package (Table 2). An important consideration is that the former only provides results of statistical pairwise comparisons, whereas the latter provides both the pairwise comparisons and the concurrent temporal overlap amongst all biological identities (e.g. three cervid species) within a null model approach.

To operate Rosario, the data were formatted as a matrix with biological identities listed in the rows (i.e. cervid species) and time intervals in the columns (i.e. 30 minutes). The width of the time intervals is determined *a priori* by users and depends on the particular study objectives and the need to establish biologically meaningful comparisons.

Refer to the vignette called "real_world_example" to see how Rosario works on a real-world scale dataset, along with a detailed explanation of the workflow (Github: https://alrobles.github.io/rosario/articles/real_world_example; R: vignette('real_world_example')). The script used to organise the data, create the time intervals and implement Rosario, activity and overlap packages is provided in the code section below. The datasets for comparison purposes needed to replicate results are available in supplementary materials (Suppl. materials 1, 2).

**Figure 3.**
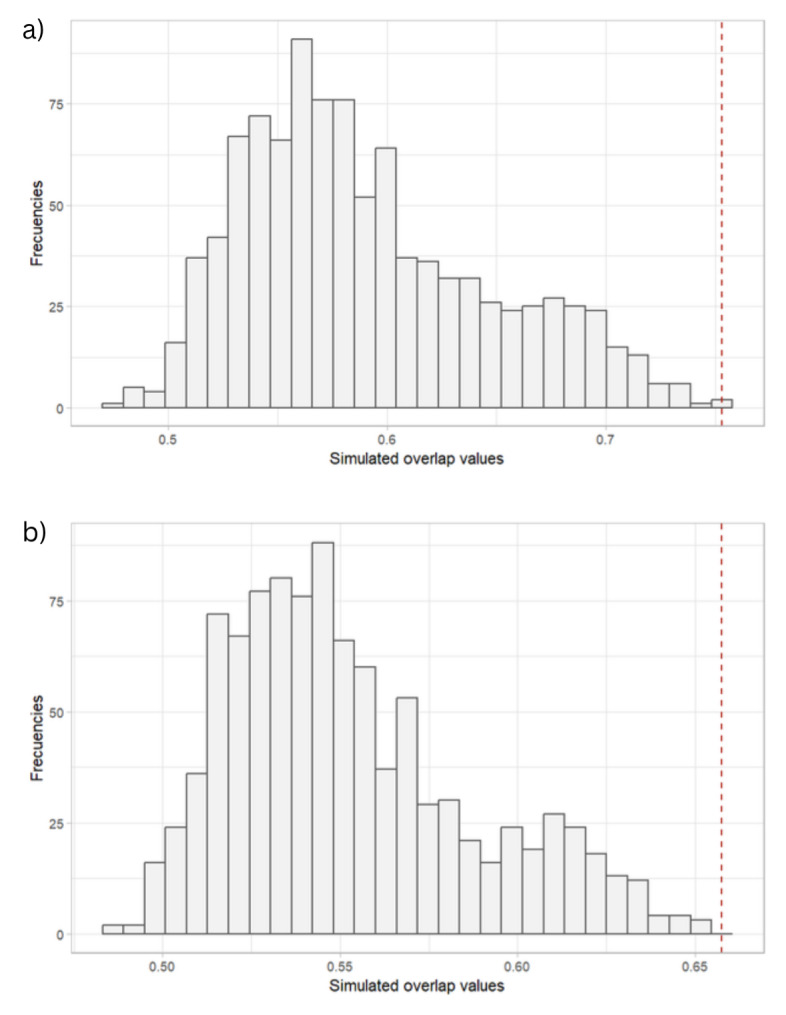
Visualisation of the observed concurrent temporal overlap (red dashed line) against the null model distribution using *temp_overlap_plot()* function a) using Pianka index; b) using Czekanowski. Both methods show that there is a higher overlap than expected by random.

After preparing the dataset, the *rescale_matrix()* function can be executed to convert the data to proportions. The *rosario()* function was used to perform randomisations of the provided data while preserving the predefined time intervals and temporal autocorrelation. This process can be visualised using the function *plot_rosario()*. Subsequently, the *temp_overlap()* function was run to calculate the concurrent temporal overlap using Pianka and Czekanowski indices amongst the three cervid’s species. The *get_null_model()* function was used to perform a null-model test of concurrent overlap via Rosario algorithm randomisations. Finally, the *temp_overlap_plot()* function was used to visualise the frequency distribution of all simulated overlap values and the observed concurrent overlap (Fig. 3).

**Figure 4.**
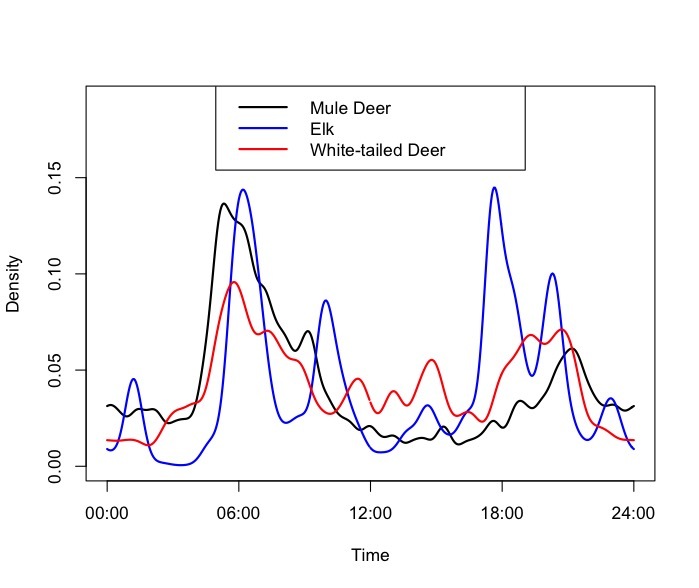
General activity patterns of three cervid species during the summer months (June - August) from 2020 - 2022 in British Columbia, Canada. Black line Mule deer, blue line Elk and red line White-tailed deer. Data were obtained from the Sim Deer project (Foster et al. 2020) on the Wildlife Insights platform.

The general activity patterns of three cervid species in British Columbia over a 24-hour period were generated using the activity package. The x-axis represents the time of day, from 00:00 to 24:00 h and the y-axis shows the density of activity. Each coloured line represents a different cervid species. Mule deer (black line) shows peaks of activity around dawn (approximately 06:00 h) and dusk (approximately 18:00 h), suggesting a crepuscular activity pattern, with additional activity observed during the night-time hours. Elk (blue line) displays a more complex pattern with a strong peak around dusk, a substantial peak during the daytime (around 10:00 h - 11:00 h) and some activity during the night. White-tailed Deer (red line) also exhibits crepuscular activity, with peaks around dawn and dusk, although the dusk peak appears less pronounced than in the Mule deer and there is noticeable activity during the middle of the day as well. In summary, all three cervid species show some level of crepuscular behaviour, with increased activity around dawn and dusk. However, they also exhibit variations in their activity levels throughout the day and night, suggesting potential differences in their ecological niches or responses to environmental factors (Fig. 4). Visual inspection does not allow for an objective determination of concurrent overlap amongst all three cervid species. Pairwise comparisons provide only a partial examination of the overlap amongst species, whereas concurrent analysis of temporal overlap amongst all cervid species can only be addressed by the null model approach implemented by Rosario.

Calculation of the activity patterns of the three cervid species through the R packages Rosario, activity and overlap, revealed that the three approaches present similar values of overlap, indicating that the overlap indices implemented by Rosario (i.e. Pianka and Czekanowski) provide comparable results to the other two packages (see Table 2). Furthermore, the use of a null model approach enables a more robust assessment of significance by comparing empirical and randomised overlap values amongst multiple biological identities.

**Code**

###Load packages ###

library(rosario)

library(dplyr)

library(lubridate)

### **Loading & preparing data** ###

Sim_dat <- read.csv("Sim_data.csv") # Data for the summer months extracted from SimDeer Project (Foster et al. 2020)

### **Helper function to bin detections for one species** ###

bin_species <- function(dat, species_code, bin_mins = 30) {

dat %>% filter(species == species_code) %>%

mutate(

timestamp = mdy_hm(timestamp), bin = floor_date(timestamp, "hour") + minutes(floor(minute(timestamp) / bin_mins) * bin_mins), hour_min_sec = format(as.POSIXct(bin), "%H:%M:%S")

) %>%

count(hour_min_sec, name = "count") %>%

tidyr::pivot_wider(names_from = hour_min_sec, values_from = count, values_fill = 0

)}

### **Create binned rows for each cervid species** ###

mule_deer <- bin_species(Sim_dat, "hemionus") # Mule deer

elk <- bin_species(Sim_dat, "canadensis") # Elk

wtd <- bin_species(Sim_dat, "virginianus") # White-tailed deer

## #**Combine species rows into the rosario input matrix** ###

binned_df <- dplyr::bind_rows(MuleDeer = mule_deer, Elk = elk, WTD = wtd, .id = "species")

### **Convert to a numeric matrix (rows = species; columns = time bins)** ###

rownames(binned_df) <- binned_df$species

data_matrix <- binned_df %>%

select(-species) %>%

mutate(across(everything(), as.numeric)) %>%

as.matrix()

rownames(data_matrix) <- binned_df$species

dim(data_matrix)

# Note: Users may analyse either counts or proportions (e.g. to compare species with very different sample sizes). The function *rescale_matrix()* rescales rows so each row sums to 1.

### **rosario package results** ###

cervid_shifts <- rosario(data_matrix[1, ]) # Create cyclic shifts and mirror images. Example: shifts for Mule deer

head(cervid_shifts)

plot_rosario(data_matrix[1, ], cols = 5) # Optional step. Example: visualise shifts for Mule deer

#Assemblage-wide temporal overlap #

Results_Pianka <-temp_overlap(data_matrix, method = "pianka")

Results_Pianka

Results_Czekanowski <-temp_overlap(data_matrix, method = "czekanowski")

Results_Czekanowski

# Null model test #

set.seed(1)

Null_Model_Pianka <- get_null_model(data_matrix, method = "pianka", nsim = 100, parallel = FALSE)

Null_Model_Pianka$p_value

Null_Model_Czekanowski <- get_null_model(data_matrix, method = "czekanowski", nsim = 100, parallel = FALSE)

Null_Model_Czekanowski$p_value

# Visualising observed concurrent temporal overlap (dashed red line) against the null distribution

temp_overlap_plot(Null_Model_Pianka)

temp_overlap_plot(Null_Model_Czekanowski)

### **activity package results** ###

# Import the data #

Sim_dat

Sim_dep <- read.csv("Sim_dep.csv") # Load deployment. Adds latitude and longitude.

Sim_dat$timestamp <- mdy_hm(Sim_dat$timestamp, tz="UTC") # Standardise to UTC if cameras do not correct for daylight savings.

Sim <- merge(Sim_dat, Sim_dep, by=c("deployment_id")) # Add deployments to data frame

# calculate solar time #

tmp <- solartime (Sim$timestamp, Sim$latitude, Sim$longitude, tz=-7, format="%Y-%m-%d %H:%M:%S")

# Although we need to use solar time, let's add both in case you want to explore the implications #

Sim$solar <- tmp$solar

Sim$clock <- tmp$clock

# Optional step: Check out the relationship between solar and clock #

plot(Sim$solar, Sim$clock)

# Species comparison #

m1 <- fitact(Sim$solar[Sim$common_name=="Mule Deer"], sample="model", reps=100) # Fit an activity model for Mule deer

m2 <- fitact(Sim$solar[Sim$common_name=="Elk"], sample="model", reps=100) # Fit an activity model for Elk

m3 <- fitact(Sim$solar[Sim$common_name=="White-tailed Deer"], sample="model", reps=100) # Fit an activity model for WTD

# Plot activity patterns into one figure #

plot(m1, yunit="density", data="none", las=3, lwd=2, tline=list(lwd=2), ylim=(c(0,0.19)))

plot(m2, yunit="density", data="none", add=TRUE, tline=list(col="blue", lwd=2), cline=list(lty=0))

plot(m3, yunit="density", data="none", add=TRUE, tline= list(col="red", lwd=2), cline=list(lty=0))

legend("top", c("Mule Deer", "Elk", "White-tailed Deer"), col=c("black", "blue", "red"), lty=1, lwd=2)

# Estimate pairwise overlap. Note repetitions reduced to speed up running time #

a <- compareCkern(m1, m2, reps = 100) # Comparison between Mule deer and Elk

b <- compareCkern(m1, m3, reps = 100) # Comparison between Mule deer and WTD

c <- compareCkern(m2, m3, reps = 100) # Comparison between Elk and WTD

# Interpret results: 0=no overlap, 1=high overlap

#### **overlap package results** ####

Sim_s<- Sim %>%

separate(timestamp, into = c("date", "time"), sep = " ")

Sim_s$time <- gettime(Sim$timestamp, "%Y-%m-%d %H:%M:%S", scale="proportion", tz="")

t_Sim_M <- 2*pi*Sim_s$time[Sim_s$common_name=="Mule Deer"] #calculating time in radians per species

t_Sim_E <- 2*pi*Sim_s$time[Sim_s$common_name=="Elk"]

t_Sim_WTD <- 2*pi*Sim_s$time[Sim_s$common_name=="White-tailed Deer"]

f_MD <- fitact(t_Sim_M, sample="data", reps=100) # Fit an activity model for Mule deer

f_E <- fitact(t_Sim_E, sample="data", reps=100) # Fit an activity model for Elk

f_WTD <- fitact(t_Sim_WTD, sample="data", reps=100) # Fit an activity model for White-tailed deer

compareAct(list(f_MD,f_E,f_WTD)) # Comparing activity patterns

# Estimate pairwise temporal overlap #

ovMD_E <- overlapEst(t_Sim_M,t_Sim_E) # Comparison between Mule deer and Elk

ovMD_WTD <- overlapEst(t_Sim_M,t_Sim_WTD) # Comparison between Mule deer and WTD

ovWTD_E <- overlapEst(t_Sim_E,t_Sim_WTD) # Comparison between Elk and WTD

## Limitations

When analysing activity data of non-syntopic biological identities, their activity or phenology needs to account for differences in time (i.e. yearly variation). For example, for diel activity, Vazquez et al. (2010) provided guidance to the potential need of time transformation depending on the location, duration of the study and latitudinal separation amongst sampling sites (Vazquez et al. 2019).

The Rosario package was specifically designed for cyclical data and is not intended for sequential (non-cyclical) datasets. However, long-term camera-trap datasets, can be analysed with Rosario when time-stamps are mapped on to any cyclical temporal domain (e.g. time of day, season or year), thereby producing a circular activity pattern suitable for the algorithm.

## Conclusions

The Rosario package generates simulated distributions of cyclical phenomena in ecology and can execute thousands of simulations while maintaining temporal autocorrelation and producing biologically realistic time-use scenarios (Castro‐Arellano et al. 2010). Originally, Rosario was designed within a restricted application framework for analysis of diel temporal overlap within a local ecological community. However, its potential for a much wider application at larger temporal and spatial scales has been recognised. Thus, transferring its functionality to a widely used popular programming language like R (R Core Team 2025) will allow for a comprehensive implementation for a larger group of users. The Rosario package analyses ecological cyclical data and is not intended for sequential datasets. However, long-term camera-trap datasets can be analysed with Rosario when time-stamps are mapped on to any cyclical temporal domain (e.g. time of day, season or year).

The primary advantage of Rosario is its ability to analyse three or more biological identities concurrently. This null model approach provides insights into the simultaneous interactions between all biological identities being analysed, which cannot be achieved by aggregating pairwise comparisons.

With the availability of Rosario in R, users now have access to a complete suite of packages within the same programming language (i.e. activity and overlap), to analyse cyclical data in ecology. These tools collectively provide a complementary perspective for understanding natural phenomena.

## Supplementary Material

61FDFE5E-060A-5FCD-9234-2752DC483FC4 10.3897/BDJ.14.e176358.suppl1 Supplementary material 1 Cervid dataset used Data type Camera trap events Brief description Dataset of cervid activity from the SIM Deer project in British Columbia, Canada, obtained from Wildlife Insights (https://app.wildlifeinsights.org) a publicly accessible dataset (Foster et al. 2020). From the over two million capture events in the SIM Deer project dataset, we focused on data collected during the summer months of June, July and August for the years 2020, 2021 and 2022. File: oo_1446128.csv https://binary.pensoft.net/file/1446128 Tatiana Velásquez-Roa (modified from Foster et al. (2020))

36CEFB92-B836-5400-B5DA-BA6DD0B058F1 10.3897/BDJ.14.e176358.suppl2 Supplementary material 2 Camera trap deployments for the Cervid dataset used Data type Camera trap deployments Brief description Camera trap deployments for the cervid dataset used in the analysis, needed to replicate results of activity and overlap packages. File: oo_1446129.csv https://binary.pensoft.net/file/1446129 Tatiana Velásquez-Roa (modified from Foster et al. 2020)

## Figures and Tables

**Table 1.** Summary of the functions implemented in the Rosario R package, descriptions of their main purposes, inputs, outputs and internal dependencies. For more detailed information, users should consult the package manual or use the built-in help documentation for individual functions. "NA" indicates that no internal dependencies are required.

**Function**	**Main purpose**	**Input(s)**	**Output**	**Uses (internal deps)**
pianka_index(p, q)	Pairwise temporal niche overlap (Pianka)	p, q: non-negative numeric vectors	Scalar in [0, 1]	NA
czekanowski_index(p, q)	Pairwise overlap (Czekanowski)	p, q: relative-frequency vector	Scalar in [0, 1]	NA
rescale_matrix(m)	Row-normalise counts to proportions	m: numeric matrix	Matrix with each row summing to 1	NA
temp_overlap_matrix(mat, method)	All pairwise overlaps amongst biological identities	mat: numeric matrix	Square symmetric matrix of overlaps	pianka_index() or czekanowski_index()
temp_overlap_df(mat)	Tidy the pairwise matrix for plotting/joins	mat: square overlap matrix (e.g., from temp_overlap_matrix)	Data frame with item1, item2, distance	stats::as.dist(), broom::tidy()
temp_overlap(mat, method)	Concurrent mean temporal overlap	mat: numeric matrix	Scalar mean of pairwise overlaps (named by method)	rescale_matrix() *(for Czekanowski)*, temp_overlap_matrix(), temp_overlap_df()
vec_permutation(numvec, x)	Cyclic rotation of a vector (start at x)	numvec: numeric vector	Rotated vector	NA
rosario(numvec)	Generate all cyclic shifts + mirrored versions (conceptual helper)	numvec: numeric vector	List of vectors (2n variants)	vec_permutation()
rosario_sample(mat)	Randomise each row by one cyclic shift; mirror with p = 0.5	mat: numeric matrix	Matrix of the same size, randomised row-wise	vec_permutation()
get_null_model(mat, method, nsim, parallel)	Null-model test of concurrent overlap via Rosario algorithm randomisations	mat: numeric matrix	List: observed_niche_overlap (scalar), p_value (htest), null_niche_overlap (data frame of simulated means)	rosario_sample(), temp_overlap(), furrr::future_map_dfr() *(if parallel)*, stats::t.test()
plot_rosario()	Diagram of the first 10 Rosario hypothetical temporal time use distributions	numvec: numeric vector	Barplot	rosario()
tem_overlap_plot()	Visualise the observed concurrent temporal overlap on the null model distribution using Pianka or Czekanowski index	numvec: numeric vector obtained from get_null_model	Histogram of simulated mean niche overlap values from a null model, overlays a dashed vertical line indicating the observed mean overlap.	get_null_model(), temp_overlap()

**Table 2.** Temporal overlap results between Mule deer, Elk and White-tailed deer (WTD) using activity, overlap and Rosario packages.

	**Activity**	**Overlap**	**Rosario**			
	Obs	Dhat4	Pianka	Observed	Czekanowski	Observed
**Mule deer vs. Elk**	0.647	0.657	0.676	0.753	0.597	0.658
**Mule deer vs. WTD**	0.766	0.755	0.861		0.742	
**Elk vs. WTD**	0.674	0.703	0.722		0.634	
